# Enhancing Multi-User Activity Recognition in an Indoor Environment with Augmented Wi-Fi Channel State Information and Transformer Architectures

**DOI:** 10.3390/s25133955

**Published:** 2025-06-25

**Authors:** MD Irteeja Kobir, Pedro Machado, Ahmad Lotfi, Daniyal Haider, Isibor Kennedy Ihianle

**Affiliations:** Department of Computer Science, Nottingham Trent University, 50 Shakespeare St., Nottingham NG1 4FQ, UK; md.kobir2023@ntu.ac.uk (M.I.K.); pedro.machado@ntu.ac.uk (P.M.); ahmad.lotfi@ntu.ac.uk (A.L.)

**Keywords:** Human Activity Recognition (HAR), Channel State Information (CSI), data augmentation, deep learning, CNN, transformer, signal processing, time-series analysis, multi-user recognition, privacy-preserving sensing

## Abstract

Human Activity Recognition (HAR) is crucial for understanding human behaviour through sensor data, with applications in healthcare, smart environments, and surveillance. While traditional HAR often relies on ambient sensors, wearable devices or vision-based systems, these approaches can face limitations in dynamic settings and raise privacy concerns. Device-free HAR systems, utilising Wi-Fi Channel State Information (CSI) to human movements, have emerged as a promising privacy-preserving alternative for next-generation health activity monitoring and smart environments, particularly for multi-user scenarios. However, current research faces challenges such as the need for substantial annotated training data, class imbalance, and poor generalisability in complex, multi-user environments where labelled data is often scarce. This paper addresses these gaps by proposing a hybrid deep learning approach which integrates signal preprocessing, targeted data augmentation, and a customised integration of CNN and Transformer models, designed to address the challenges of multi-user recognition and data scarcity. A random transformation technique to augment real CSI data, followed by hybrid feature extraction involving statistical, spectral, and entropy-based measures to derive suitable representations from temporal sensory input, is employed. Experimental results show that the proposed model outperforms several baselines in single-user and multi-user contexts. Our findings demonstrate that combining real and augmented data significantly improves model generalisation in scenarios with limited labelled data.

## 1. Introduction

Human Activity Recognition (HAR) is a foundational task in ubiquitous computing, offering critical insights into human behaviour with broad applications in healthcare monitoring, smart environments, ambient assisted living, and elder care [1]. Traditional HAR systems typically rely on ambient sensors, wearable sensors or vision-based systems. Although effective, these modalities pose considerable limitations; ambient sensors such as motion detectors do not provide accurate information about a specific activity, wearable devices demand user compliance, regular charging, and maintenance, while vision-based systems suffer from occlusion, varying illumination, and severe privacy concerns [2,3,4]. To preserve the privacy of users, some research with thermal vision employing thermal sensor arrays has shown promising results [5].

To address these limitations, recent advances have led to device-free HAR using wireless sensing, particularly Wi-Fi Channel State Information (CSI). CSI captures fine-grained changes in radio signal propagation due to human motion, enabling passive, contactless, and privacy-preserving activity detection [6]. The advantage of this approach lies in the sensing through obstacles and across rooms without requiring user-worn devices. However, CSI-based HAR systems face challenges including sensitivity to ambient noise, temporal drift, and multipath interference. Performance often degrades in dynamic or densely populated multi-user environments due to overlapping activities and spatial interference [7]. The limited availability of labelled datasets, especially for diverse user configurations and rare activities, hinders the training of generalisable models [8]. Multi-user HAR is further complicated by simultaneous movements, unpredictable patterns, and spatial entanglement, requiring models capable of extracting discriminative features from noisy, entangled signals and generalising across varying spatial configurations [9,10,11]. Traditional machine learning techniques, relying on handcrafted features and rigid assumptions, are often inadequate in these contexts [12]. Deep learning, particularly convolutional and attention-based models, offers an alternative. However, many current studies treat preprocessing, feature extraction, and classification as discrete stages, potentially missing the benefits of an integrated pipeline.

Another inherent challenge associated with CSI-based HAR is data scarcity, which significantly limits the training of deep learning models, especially in complex multi-user and real-world scenarios where manual annotation is inefficient. Data augmentation provides a viable strategy to address this and improve model robustness [12,13]. Unlike image augmentation, time-series augmentation for CSI data must preserve signal continuity, statistical integrity, and temporal dynamics. Techniques like jittering (Gaussian noise), scaling (amplitude modulation), magnitude warping (non-linear time scaling), and slice shuffling (temporal segment permutation) generate realistic signal variations mimicking unseen behaviours or environments [14]. The application of these augmentations increases data diversity and enhances the generalisation of models trained with limited data.

Recent advances in deep learning highlight the potential of hybrid architectures combining convolutional layers for local pattern recognition with Transformers for capturing long-range dependencies [11,15,16,17]. However, their multi-user application, device-free HAR, remains largely unexplored. Transformers, particularly when augmented with relative positional encoding, are well-suited for modelling the complex temporal relationships inherent in overlapping or concurrent human activities [18]. To address these limitations, a unified, augmentation-aware framework for CSI-based HAR is proposed, specifically designed to handle the challenges of multi-user recognition and limited training data. Our approach integrates signal denoising, advanced data augmentation, domain-informed feature engineering, and deep multimodal learning. As part of this, a custom CNN + Transformer model is introduced to learn both local and global patterns in the CSI signal space. It is trained using an augmented dataset mimicking real-world activity variability.

The scientific contributions resulting from the research presented in this paper are as follows:

- An integrated CSI preprocessing and augmentation pipeline is introduced, combining Butterworth filtering, Discrete Wavelet Transform (DWT), and random transformation strategies to augment and enrich training diversity while preserving statistical properties.
- A custom integration of CNN and Transformer components for effective local and global temporal feature extraction, specifically adapted for multi-user activity recognition using augmented CSI data.
- The proposed architecture is benchmarked against three state-of-the-art hybrid models, demonstrating better performance and generalisability across varying augmentation levels and user concurrency.
- Extensive experiments to evaluate different multi-user scenarios are conducted, demonstrating the effectiveness of the proposed model in mitigating signal overlap and spatial interference.

The remainder of the paper is organised as follows: Section 2 presents a review of related work, while Section 3 details the proposed model and augmentation methods. Section 4 describes the experimental setup and results, followed by discussion in Section 5. The paper concludes in Section 6, offering final reflections and directions for future research.

## 2. Related Work

Advances in HAR using CSI have seen a notable shift from conventional machine learning methods to advanced deep learning models, with Transformer-based architectures emerging as a powerful alternative in recent years. This is driven by the increasing need for scalable, device-free, and privacy-conscious activity recognition systems, especially in smart homes and IoT-enabled environments [1,4,6,7]. Classical machine learning algorithms like Support Vector Machines (SVM) and Random Forests (RF) have inherent limitations in feature generalisation and sensitivity to noise, prompting a transition toward deep learning models [7,17]. CNN architectures learn spatial representations directly from spectrograms of CSI signals, outperforming hand-engineered features [4,6,7,17]. To enhance temporal modelling for capturing sequential dependencies in activities, hybrid architectures have been utilised by combining CNN architectures with recurrent models such as LSTM and GRU. Recent approaches have also explored contrastive learning frameworks and diffusion-based methods for HAR, which learn robust representations by maximising inter-class separability and modelling temporal dynamics in latent space [19,20]. Diffusion-based techniques have also been proposed for data augmentation and sensing enhancement in Wi-Fi-based HAR, offering generative mechanisms for more realistic and diverse training samples [21,22]. Although these methods show promise, our work focuses on supervised learning with architectural emphasis on CNN and Transformer integration to support interpretability and scalability in multi-user environments.

Advances in Transformer-based architectures are emerging in CSI-HAR by incorporating self-attention to capture long-range temporal dependencies, offering a robust alternative to recurrence [11,15,16]. For example, a multichannel attention-based Transformer achieved high accuracy in HAR [23], and a lightweight Transformer optimised for edge computing maintained a good performance—92.4% accuracy with reduced complexity [24]. However, attention-only models can struggle with fine-grained local features to discriminate similar activities. To overcome this, hybrid CNN–Transformer architectures have emerged, combining CNNs for spatial pattern extraction with Transformers for global contextual modelling. Incorporating relative positional embeddings in these frameworks enhances temporal precision and activity segmentation [23,25]. These hybrid designs improve recognition accuracy and balance local and global sequence modelling, often a trade-off in Transformer-only systems.

Table 1 presents a summary of recent advances in CSI-HAR, showcasing a range of datasets, modelling techniques, signal processing approaches, and evaluation protocols. The field has evolved significantly from classical machine learning models like SVM and Random Forest to more adaptive and accurate deep learning architectures such as CNNs [26], attention-based BiLSTM models [27], and compact Transformer-based models. More recent efforts also focus on hybrid frameworks that address domain variability through domain adaptation and generalisation [28,29].

A recurring challenge across many studies is the limited generalisability of models in unseen or changing environments. To address this, several works have proposed strategies like few-shot learning [31,32], one-shot recognition [35], and zero-effort cross-domain adaptation [34]. These methods aim to reduce the reliance on large, curated training datasets, enabling models to adapt more flexibly to novel contexts or user behaviours.

Another major focus is data augmentation. Diffusion-based methods [33] have emerged as a powerful way to create more realistic synthetic CSI samples, often in combination with contrastive learning to improve performance under limited supervision. These approaches have shown promise in boosting accuracy and resilience, especially when training data is scarce or noisy.

Despite these improvements, recognising activities in multi-user environments remains a complex problem. Overlapping signals often reduce model accuracy, even when advanced techniques are used to separate them. Abuhoureyah et al. [30] tackled this by applying independent component analysis (ICA) and continuous wavelet transform (CWT), demonstrating improved performance for location-independent, multi-user HAR. However, such solutions are still sensitive to dynamic spatial configurations and signal interference.

Finally, evaluation inconsistency remains a barrier to progress. Studies vary significantly in their methodologies, with some using 10-fold cross-validation [27], or contrastive frameworks [33]. This lack of standardisation makes it difficult to compare models directly or replicate published results reliably.

In light of these ongoing challenges, our proposed work introduces a hybrid CNN + Transformer framework that is designed with generalisability and scalability in mind. By combining spatial–temporal feature extraction with efficient preprocessing and augmentation strategies, the model aims to deliver robust performance in both single- and multi-user scenarios, with real-world applicability at its core.

## 3. Proposed Methods

**Figure 1:**
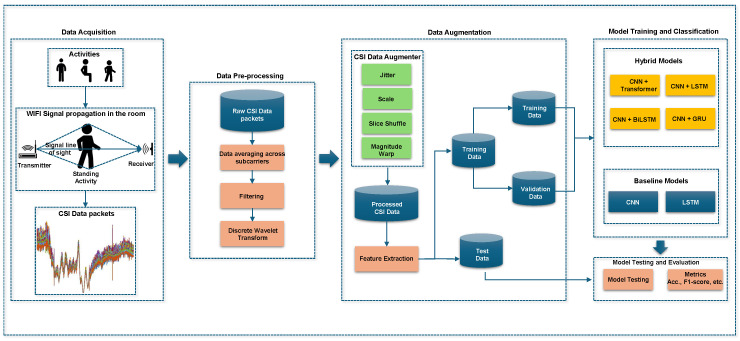
Overview of the proposed Human Activity Recognition framework using CSI data. The pipeline consists of four main stages: data acquisition through Wi-Fi signal propagation, signal preprocessing using filtering and wavelet transformation, data augmentation via random transformation techniques (jittering, scaling, slice shuffling, and magnitude warping), and classification using both baseline and hybrid deep learning models.

A four-stage approach for HAR using CSI is proposed. The schematic diagram of the proposed framework is illustrated in Figure 1, designed to enhance recognition performance, particularly in complex multi-user home environments. The pipeline of the proposed approach comprises data acquisition, preprocessing, augmentation, and model training/classification. The data acquisition involves transmitting Wi-Fi signals through an indoor space where human activities occur. Motion-induced perturbations of these signals are captured as CSI measurements at the receiver, inherently encoding characteristics of static postures and dynamic movements, forming the raw dataset.

A multi-stage preprocessing pipeline extracts meaningful information from raw CSI. Subcarrier averaging reduces redundancy and smooths signal fluctuations. Butterworth filtering eliminates high-frequency noise, and Discrete Wavelet Transform (DWT) decomposes the signal, preserving essential features across scales, improving signal quality and retaining informative temporal dynamics. To improve generalisability and reduce overfitting given limited CSI data, a random transformation-based augmentation module generates diverse and realistic variations while preserving activity class semantics. This module incorporates jittering, scaling, slice shuffling, and magnitude warping. The augmented dataset is then partitioned for model development.

The classification stage trains baseline (CNN, LSTM) and advanced hybrid deep learning models CNN with Transformer - the proposed model, BiLSTM, GRU and LSTM to better capture spatial and temporal dependencies inherent in CSI data, enhancing recognition of complex, multi-user activities due to their increased representational capacity. Finally, the trained models are evaluated using accuracy and F1-score metrics to assess their effectiveness and robustness.

### 3.1. Data Acquisition

A publicly available CSI-based HAR dataset [36], specifically designed to investigate the feasibility of indoor activity recognition using fine-grained CSI from wireless signals, is utilised. The dataset was collected in a controlled laboratory environment, simulating a typical indoor room measuring 3 m by 2.8 m.

**Figure 2:**
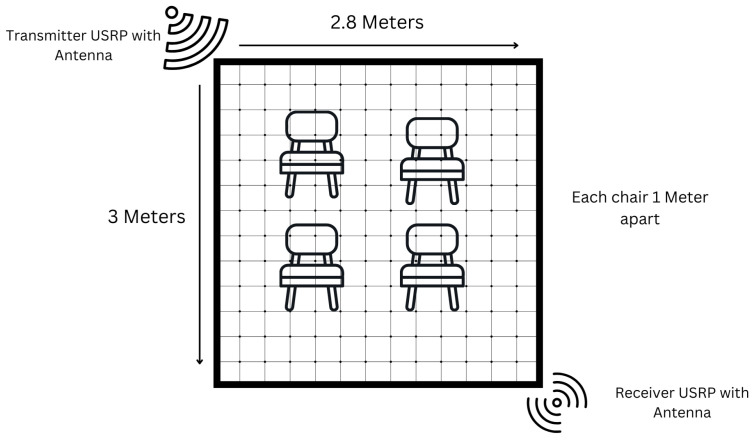
Experimental setup for capturing human activities using the 5G frequency band. The indoor environment measures 3 m by 2.8 m. A USRP X300 (transmitter) and a USRP X310 (receiver) are positioned at opposite corners. Four chairs are arranged 1 m apart to define the human activity space.

As illustrated in Figure 2, the experimental setup involved two Universal Software Radio Peripheral (USRP) devices: a USRP X300 as the transmitter and a USRP X310 as the receiver. Both were equipped with VERT2450 omnidirectional antennas and operated at a frequency of 3.75 GHz within the 5G sub-6 GHz band, chosen for its ability to capture subtle human motion with low interference and high signal resolution. The transmitter and receiver were positioned diagonally opposite each other in the room to establish a reliable communication link and maximise spatial signal coverage. Within the defined activity zone, consisting of four chairs arranged in a 1 m grid, participants performed various daily activities. During these activities, the USRP devices continuously collected CSI data using the GNU Radio software environment. The communication protocol used Orthogonal Frequency-Division Multiplexing (OFDM), providing robust frequency-domain information. The raw CSI data was initially logged in plain text format and subsequently converted to CSV files for preprocessing and machine learning analysis [36].

### 3.2. Data Processing

To ensure accurate HAR, a data processing pipeline is designed to denoise raw CSI streams and extract meaningful time–frequency features. A Butterworth low-pass filter was applied first to eliminate high-frequency noise. Subsequently, a DWT was used to extract hierarchical temporal–frequency components, capturing subtle variations induced by human activity. This dual-stage approach effectively handles both single-user and multi-user scenarios, where overlapping motion patterns often introduce complex signal artifacts [24,37]. The initial step involved averaging the raw CSI data across all 51 subcarriers to standardise the input and reduce variability, resulting in a single representative time series $\bar{x}\left(t\right)$, as defined by Equation (1):

(1) $$\bar{x}\left(t\right)=\frac{1}{N}\sum_{i=1}^{N}x_{i}\left(t\right),\;\mathrm{where}\;N=51$$

**Figure 3:**
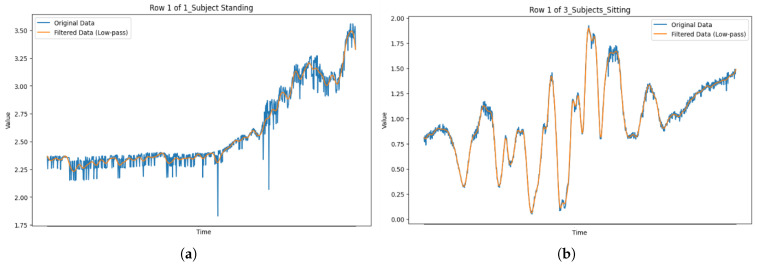
Comparison of original and Butterworth-filtered CSI signals for single-user and multi-user activity sessions. (**a**) One subject standing, (**b**) three subjects sitting.

To mitigate high-frequency noise originating from hardware, environmental factors, and multipath effects, which can distort activity-related dynamics, a fourth-order Butterworth low-pass filter was applied. The maximally flat passband response enables effective noise suppression while preserving critical signal patterns, with the denoised signal $\tilde{x}\left(t\right)$ obtained through zero-phase filtering:

(2) $$\tilde{x}\left(t\right)=F_{\mathrm{LP}}\left[\bar{x}\left(t\right)\right],$$

where $F_{\mathrm{LP}}$ denotes the zero-phase Butterworth filtering operation, which avoids phase shifts that might distort time-domain features. Figure 3 illustrates the filtering effect on CSI signals for single- and multi-user activities.

While the Butterworth filter reduces noise, it does not capture the non-stationary and transient nature of human motion. Therefore, the denoised signal $\tilde{x}\left(t\right)$ was further processed using the DWT. This technique decomposes the signal into time–frequency components across multiple scales, extracting rich hierarchical features.

Using the Haar wavelet ψ(t) and decomposition level *j*, the DWT coefficients $W_{j,k}$ were computed as

(3) $$W_{j,k}=\int \tilde{x}\left(t\right)\cdot \psi_{j,k}\left(t\right)\;dt,$$

where $\psi_{j,k}\left(t\right)=2^{-j/2}\psi \left(2^{-j}t-k\right)$ represents the scaled and translated mother wavelet. A decomposition level of J=3 was selected to balance detail extraction and computational cost. This produced approximation coefficients $A_{3}$ (low-frequency structure) and detail coefficients $D_{1}$, $D_{2}$, and $D_{3}$ (high-frequency transitions). These coefficients formed the basis for subsequent feature extraction and classification.

### 3.3. CSI Augmentation Techniques

Real-world CSI datasets for HAR often exhibit limitations in size and suffer from class imbalance. These characteristics pose significant challenges for training deep learning models, frequently leading to overfitting and reduced classification performance, particularly for underrepresented activity classes. To address these limitations and improve model generalisation, a data augmentation pipeline based on random transformation strategies was implemented. This approach aims to generate diverse training instances that effectively simulate the inherent variability of real-world CSI signals while preserving the key discriminative features associated with each activity, thereby enhancing both model robustness and its ability to generalise to unseen data [38].

Let $x=(x_{1},x_{2},\ldots ,x_{T})$ denote a processed CSI data frame, where each $x_{t}\in R^{n}$ represents the amplitude vector across *n* subcarriers at a specific time step *t*, and *T* is the total temporal window size of the data frame. The augmentation process then produces a transformed sequence $x^{\prime }=A\left(x\right)$, where A represents a specific transformation operator applied to the original CSI data frame x. In our augmentation pipeline, a combination of time-series augmentation methods informed by prior research in HAR and time-series analysis is employed: jittering, scaling, slice shuffling, and window warping. The specific implementations and parameters for these transformations were adapted from established techniques and findings in the literature [39,40].

### 3.4. CSI Augmentation Techniques

Real-world CSI datasets for HAR often suffer from limited size and class imbalance. These characteristics pose significant challenges for training deep learning models, frequently leading to overfitting and reduced classification performance, especially for underrepresented activity classes. To mitigate this, a data augmentation pipeline based on random transformation strategies is implemented. This approach generates diverse training instances that simulate real-world signal variability while preserving key discriminative features, thereby enhancing model generalisation and robustness [38].

Let $x=(x_{1},x_{2},\ldots ,x_{T})$ denote a processed CSI data frame, where each $x_{t}\in R^{n}$ represents the amplitude vector across *n* subcarriers at time step *t*, and *T* is the temporal window size. The augmentation process produces a transformed sequence $x^{\prime }=A\left(x\right)$, where A is a transformation operator. Multiple time-series augmentation techniques are incorporated. This include jittering, scaling, slicing, and window warping, each designed to simulate different real-world signal distortions while maintaining temporal coherence [39,40]. Jittering introduces small Gaussian noise perturbations to simulate sensor noise. Scaling adjusts the amplitude dynamics to reflect variations in user strength or distance. Slicing randomly extracts sub-segments of the CSI window to improve robustness against partial observations, and window warping modifies the temporal progression to mimic speed variations in activity execution. Collectively, these augmentations expand the diversity of the training data, aiding the model in learning more invariant and generalisable representations.

**Jittering**: Gaussian noise is added to simulate sensor or environmental interference. Given a noise vector $\epsilon ∼N(0,\sigma^{2})$, the augmented sequence becomes

(4) $$x^{\prime }=x+\epsilon .$$

**Scaling**: A random scalar factor α∼U(a,b) adjusts the amplitude to emulate variations in movement intensity or proximity to the transmitter:

(5) $$x^{\prime }=\alpha \cdot x.$$

**Slicing**: To simulate temporal disorder, a segment $x_{k:k+m}$ is repositioned in the sequence. This maintains class semantics while introducing variability:

(6) $$x^{\prime }=\mathrm{concat}\left(x_{1:k},x_{k+m:T},x_{k:k+m}\right).$$

**Window Warping**: A non-linear warping function ϕ(t) temporally stretches or compresses a windowed segment to reflect changes in human motion speed:

(7) $$x_{t}^{\prime }=x_{\phi (t)}.$$

**Figure 4:**
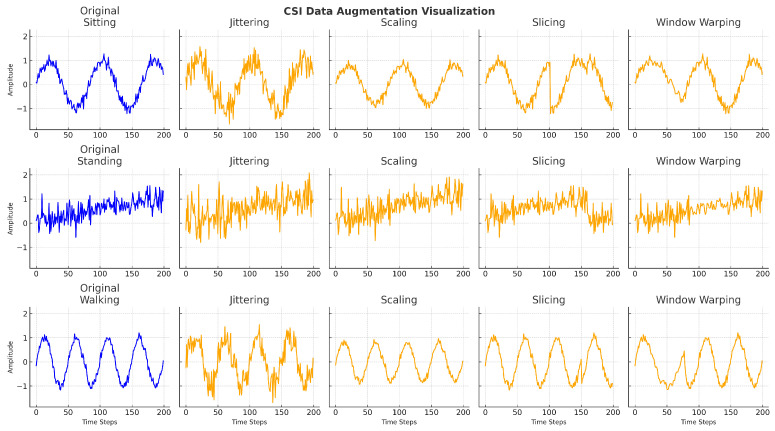
Augmentation impact of random-transformation techniques on CSI time-series for different activities. Each row displays the original and augmented signals using jittering, scaling, slicing, and window warping, respectively.

The effects are visualised in Figure 4. Each augmentation technique introduces controlled intra-class variability while preserving the core temporal structure essential for accurate recognition. This controlled diversity enhances the ability of the model to generalise to unseen activity patterns and user behaviours. To ensure label consistency, the augmentation techniques were applied within bounded transformations that retain the temporal and structural characteristics of the original signal, thereby preserving class identity across augmented samples.

### 3.5. Feature Extraction

Our feature extraction strategy integrates time-domain, frequency-domain, and entropy-based measures to comprehensively represent both macroscopic motion trends and subtle signal variations crucial for HAR [41,42,43]. In total, 34 features are extracted from the preprocessed CSI signals to capture the underlying physical and spectral characteristics of human motion across a diverse range of activity classes.

**Time-Domain Features**: Statistical properties of the CSI signal within short temporal windows are computed to detect gross motion transitions. Metrics such as mean, variance, standard deviation, skewness, kurtosis, and root mean square (RMS) reflect signal magnitude and variability, essential for distinguishing static from dynamic states [42]. For instance, RMS indicates activity intensity, effectively differentiating walking (high RMS) from sitting (low RMS).

**Frequency-Domain Features**: Spectral characteristics using Fast Fourier Transform (FFT) techniques are used to capture periodicity and energy distribution. These include spectral entropy (quantifying frequency content unpredictability), spectral centroid (indicating dominant frequency band), and specific FFT coefficients, instrumental in identifying cyclic patterns in repetitive activities like walking, running, or cycling [43].

**Entropy-Based Features**: Permutation entropy and its weighted variant to quantify signal complexity and irregularity are incorporated, measuring the temporal unpredictability and structural complexity of CSI waveforms [41,42]. These are valuable for distinguishing subtle postural variations or transitions lacking strong spectral or amplitude signatures, such as differentiating standing from leaning based on their distinct temporal dynamics.

The integration of these features provides a holistic CSI signal representation, capturing magnitude, frequency distribution, and temporal complexity. A detailed breakdown is in Table 2. The feature set also includes advanced descriptors like wavelet energy, power bandwidth, and spectral distance, further enriching the feature space and supporting improved model generalisation across diverse activity contexts.

### 3.6. The Proposed CNN + Transformer Model

HAR from CSI data involves mapping complex, high-dimensional time-series inputs to discrete activity classes. To guide the architectural design and learning approach, we first provide a mathematical formulation of the underlying classification task. Let $D={\left\{\left(x^{\left(i\right)},y^{\left(i\right)}\right)\right\}}_{i=1}^{N}$ be a set of *N* preprocessed and feature-extracted CSI samples, where $x^{\left(i\right)}\in R^{d}$ denotes the *d*-dimensional input feature vector and $y^{\left(i\right)}\in \left\{1,2,\ldots ,K\right\}$ is the corresponding activity label among *K* possible classes. The goal is to learn a function $f_{\theta }:R^{d}\to \left\{1,\ldots ,K\right\}$ parameterised by θ, which minimises a classification loss over the dataset:

(8) $$\begin{array}{c} \min_{\theta }\;\frac{1}{N}\sum_{i=1}^{N}L\left(f_{\theta }\left(x^{\left(i\right)}\right),y^{\left(i\right)}\right), \end{array}$$

where L is the cross-entropy loss function appropriate for multi-class classification.

The preprocessing pipeline aims to enhance feature separability by suppressing irrelevant signal variations, including hardware-induced noise and emphasising activity-related patterns. Subcarrier averaging reduces dimensionality and smooths spatial fluctuations. Butterworth filtering attenuates high-frequency noise, preserving essential motion-induced signal changes. The DWT further decomposes signals into localised time–frequency representations, which increases the discriminative power of extracted features, particularly important for distinguishing between static (e.g., sitting, standing) and dynamic (e.g., walking) activities. This preprocessing improves the signal-to-noise ratio, enhancing inter-class distances and stabilising intra-class variance.

**Figure 5:**
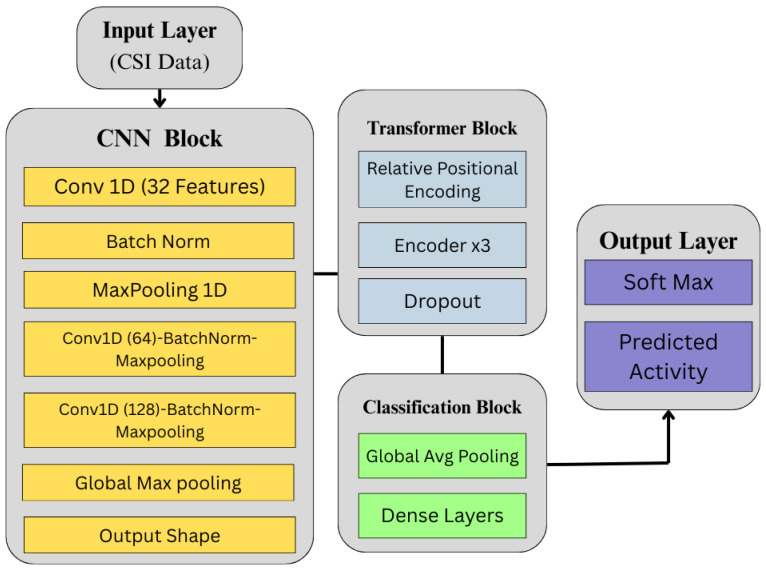
Architecture of the proposed time-series analysis model, incorporating CNN for feature extraction and a Transformer encoder for temporal representation, followed by a classification block.

Our model architecture, depicted in Figure 5, uses a hybrid deep learning framework combining CNN and Transformer encoders for effective local and global feature extraction from time-series CSI data. This design captures the complex temporal features of HAR tasks by taking advantage of the CNN layers to learn local dependencies and the Transformer block for long-range contextual relationships [44,45]. See also Table 3 for the hyperparameter settings of the proposed CNN + Transformer model. The model consists of three components: a CNN layer for feature extraction, a Transformer block for relative positional encoding, and a classification head. This is denoted as

(9) $$\begin{array}{cc} X & =\left\{x_{0},x_{1},x_{2},\ldots ,x_{T}\right\},\;x_{t}\in R^{d}, \end{array}$$

where each instance contains *T* time steps and *d* features per step. For univariate processing, the input is reshaped to (T,1).

**CNN Feature Extractor Block.** This block comprises three 1D convolutional layers. The first layer applies 32 filters with a kernel size of 3, followed by Batch Normalisation and MaxPooling1D with a pool size of 2. The second and third convolutional layers use 64 and 128 filters, respectively, and include similar normalisation and pooling. ReLU activation is applied throughout:

(10) $$\begin{array}{cc} f(x) & =\max(0,x), \end{array}$$

The output is passed through Global Max Pooling and reshaped to (1, 128) to match the input expected by the Transformer.

**Transformer Encoder Block.** To preserve temporal sequence information, vector-based relative positional encoding is applied:

(11) $$\begin{array}{cc} X_{\mathrm{pos}} & =X+E_{\mathrm{pos}},\;E_{\mathrm{pos}}\in R^{L\times d}, \end{array}$$

where *L* is the sequence length. The Transformer comprises three encoder layers, each with a multi-head self-attention mechanism:

(12) $$\begin{array}{cc} \mathrm{Attention}(Q,K,V) & =\mathrm{softmax}\left(\frac{QK^{⊤}}{\sqrt{d_{k}}}\right)V, \end{array}$$

Each encoder includes residual connections, Layer Normalisation, and feedforward layers implemented with 1D convolutions of size (256,d) using ReLU activation.

**Classification Head.** The Transformer output is passed through a Global Average Pooling layer and two dense layers with 256 and 128 units, respectively. ReLU is used along with Dropout (0.5 and 0.3) to reduce overfitting. The final output layer uses softmax activation:

(13) $$\begin{array}{c} \hat{y}=\mathrm{softmax}\left(Wx+b\right), \end{array}$$

This architecture provides an optimal balance between representational power and computational efficiency, making it particularly suitable for deployment in real-time or edge-based HAR systems [44].

### 3.7. Performance Measurement Criteria

To evaluate our CNN + Transformer model, accuracy and F1-score metrics are chosen to address the potential class imbalance prevalent in CSI-based HAR datasets. While accuracy represents the proportion of correct classifications, it can provide a skewed view in imbalanced scenarios. F1-score, the harmonic mean of precision and recall, and specificity (true negative rate) to obtain a more balanced performance assessment are also utilised. The mathematical definitions of these metrics are as follows:

(14) $$\begin{array}{cc} \mathrm{Accuracy} & =\frac{TP+TN}{TP+TN+FP+FN} \end{array}$$

(15) $$\begin{array}{cc} \mathrm{Precision} & =\frac{TP}{TP+FP} \end{array}$$

(16) $$\begin{array}{cc} \mathrm{Recall} & =\frac{TP}{TP+FN} \end{array}$$

(17) $$\begin{array}{cc} \mathrm{F1-Score} & =2\cdot \frac{\mathrm{Precision}\cdot \mathrm{Recall}}{\mathrm{Precision}+\mathrm{Recall}} \end{array}$$

Model generalisation was evaluated using 10-fold cross-validation. The performance results are then averaged across the ten folds to provide a reliable estimate of the model’s ability to perform on unseen data.

## 4. Experiments and Results

This section details the experimental setup and presents the results obtained to evaluate the effectiveness and robustness of our proposed CSI-based HAR framework. A thorough analysis of the performance of the novel CNN + Transformer model is provided in comparison to several established baseline architectures. The experiments were structured to assess key aspects of our approach, including the impact of data augmentation, the model’s performance in multi-user scenarios, and its overall classification accuracy across different activity classes.

The experiments were conducted in a Python 3.12 environment utilising the TensorFlow backend with Keras, with the Scikit-Learn, NumPy, and Pandas libraries for data manipulation and evaluation. Following the processing of the raw CSI data, the resulting dataset comprised instances representing the subcarriers across different channels. The original dataset contained 1777 instances. For each activity and user, 34 columns represented the extracted features, as detailed in Section 3.

### 4.1. Experiment I: Sensitivity Analysis of Augmented vs. Original CSI Data

To evaluate the impact of data augmentation on the statistical properties of the original CSI dataset, a sensitivity analysis is conducted comparing the distributions of extracted features, specifically, mean values across multiple multi-user activity configurations. The main objective of this experiment was to determine whether the augmentation process introduces statistically significant deviations in the distributional characteristics of CSI features. To this end, the following hypothesis is formulated:

- Null Hypothesis ($H_{0}$): The distributions of the original and augmented data are statistically identical, i.e., augmentation does not significantly alter feature distributions.
- Alternative Hypothesis ($H_{1}$): The distributions of the original and augmented data differ significantly.

To determine the appropriateness of parametric versus non-parametric testing, initially performed normality tests. The test was performed on both the original and augmented datasets using the Shapiro–Wilk (SW) [46] and Anderson–Darling (AD) tests [47]. For example, the SW test results for the two subjects (one sitting and one standing) class where W = 0.9842, p=0.2795 for the original data and W = 0.9885, $p=1.425\times 10^{-8}$ for the augmented data. Similarly, the AD test returned A^2^ = 0.5591 for the original and A^2^ = 7.0680 for the augmented data both exceeding critical thresholds. These results suggest a violation of the normality assumption, especially for the augmented data. Therefore, non-parametric statistical tests were chosen.

The Mann–Whitney U test was used to examine whether there were statistically significant differences in the means of the two datasets. Levene’s test was also used to assess the homogeneity of variances. As summarised in Table 4, the *p*-values from both tests exceeded 0.05 for all classes, indicating no statistically significant differences in central tendency or variance. Thus, we fail to reject the null hypothesis in all cases.

**Figure 6:**
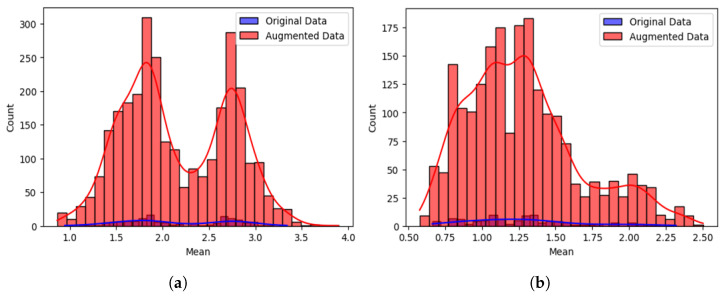
Sensitivity analysis comparing the distribution of mean values between original and augmented CSI data for two multi-user activity scenarios: (**a**) two subjects performing, one sitting and one standing, and (**b**) three subjects performing, two sitting and one standing. Augmented data (red) show broader tails but remain centred similarly to original data (blue): (**a**) two subjects, one sitting and one standing (**b**) three subjects, two sitting and one standing.

To complement these statistical findings, visualisations of the feature distributions were included. Figure 6 shows histograms for the two subjects (one sitting and one standing) and three subjects (two sitting and one standing) classes. While the augmented data (in red) displays broader tails, the distributions remain centred around the same mean as the original data (in blue), suggesting that augmentation preserves core statistical structure.

**Figure 7:**
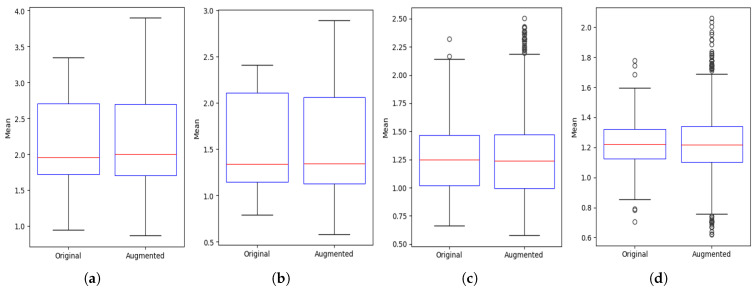
Boxplot comparison of mean values between original and augmented CSI data across multi-user activity scenarios. Augmentation maintains central tendency and spread while introducing slight variations, especially with increased subject count: (**a**) one subject sitting (**b**) two subjects, one sitting and one standing; (**c**) three subjects, two sitting and one standing; (**d**) four subjects, two sitting and two standing.

In Figure 7, the boxplots for four activity configurations are presented, comparing spread, median, and outliers. The plots confirm that augmentation maintains the original data’s distributional characteristics, with only minor variability introduced in higher-subject configurations.

### 4.2. Experiment II: Impact of Varying Augmentation Factors Across Models

This experiment investigates the influence of different data augmentation factors on the performance of the proposed CNN + Transformer model and three baseline hybrid architectures: CNN + BiLSTM, CNN + GRU, and CNN + LSTM. The data augmentation techniques detailed earlier to expand the models’ decision boundaries were applied, aiming to enhance their generalisation capability on the original dataset and yield more efficient classifiers. A range of augmentation factors was experimented with to determine their optimal impact. For example, applying an augmentation factor of 3 increased the original dataset size from 1777 instances to 5331 instances (1777 multiplied by 3) for each augmentation method.

Table 5 presents the accuracy scores obtained for augmentation factors ranging from 0 to 10. The results demonstrate a consistent performance improvement from factor 1 to factor 5 across all evaluated models and activity phases, highlighting the benefit of moderate data augmentation. For example, the CNN + Transformer model’s accuracy increased from 0.757 (no augmentation) to 0.939 at an augmentation factor of 5, while the CNN + GRU model’s accuracy rose from 0.772 to 0.930 under the same conditions. It is noted that beyond an augmentation factor of 5, the performance gains begin to plateau, as illustrated by the CNN + LSTM model and accuracy increased only marginally from 0.927 at factor 5 to 0.959 at factor 10. This observation aligns with the principle of diminishing returns often associated with excessive data augmentation [38]. Furthermore, none of the models experienced a performance decline even at the highest tested augmentation factor of 10, underscoring the robustness of the specific augmentation techniques employed. The consistently lower performance observed at an augmentation factor of 0 (i.e., without any augmentation) further emphasises the critical role of data augmentation in enhancing the performance of deep learning models for HAR tasks, particularly when dealing with datasets that may be sparse or exhibit class imbalance.

### 4.3. Experiment III: Multi-User Presence and Activity Detection

This experiment evaluated activity recognition performance in multi-user scenarios (ranging from one to four concurrent users, including a mixed-user setting). Table 6 presents classification results for each model. High performance was observed across all models in the single-user setting, with the CNN + Transformer achieving an F1-score of 0.997. As the number of users increased, a marginal decline in performance was noted, particularly in the four-user configuration.

The CNN + Transformer model demonstrated very good performance with an F1-score of 0.934 in the mixed multi-user scenario, highlighting its effective use of attention mechanisms for distinguishing activity signals. CNN + BiLSTM and CNN + GRU performed comparably in the two- and three-user settings, while CNN + LSTM showed a more noticeable performance drop in the four-user and mixed scenarios (F1-score: 0.926). These results underscore the necessity for advanced temporal and attention-based models to manage user concurrency, aligning with trends in ambient intelligence and ubiquitous computing [48].

## 5. Discussion

The experimental results demonstrate the performance of the proposed CSI-based HAR approach across various setups, highlighting its capability to handle data diversity and complex multi-user environments. This can be largely attributed to the integrated pipeline of signal preprocessing, data augmentation, hybrid deep learning architecture, and comprehensive evaluation. The following key aspects of the system are further discussed:

### 5.1. Influence of Augmentation Factor on Performance

Experiment II explored the effect of varying augmentation factors, providing insights into how training diversity influences generalisation. All models showed improved accuracy with augmentation factors up to 5, with CNN + Transformer experiencing the highest increase from 0.757 to 0.939. This supports prior research indicating that moderate augmentation mitigates overfitting and enhances robustness [38,49]. Performance plateaued beyond factor 5, suggesting diminishing returns due to possible redundancy or over-perturbation [38]. Importantly, there was no decline in accuracy at factor 10, confirming the soundness of the augmentation strategy and its ability to preserve signal semantics. Specifically, the results also show the effectiveness of other techniques as jittering, slice shuffle, scaling and magnitude warping, in enhancing the overall performance. To further understand the contribution of individual augmentation techniques, additional experiments are conducted, the results of which are presented in Table 7. This table shows the performance of the CNN + Transformer model (at an augmentation factor of 5) with different combinations of our augmentation strategies.

Interestingly, while magnitude warping alone led to a noticeable drop in performance (Table 7), its inclusion within our complete augmentation pipeline contributed positively when combined with other techniques. This suggests that although aggressive or poorly controlled warping can distort temporal dynamics and impede learning, the combined effects, when balanced with jitter, scaling and slice shuffle, can enhance data variability without compromising the underlying signal integrity. This finding underscores the importance of carefully designing and balancing augmentation strategies rather than relying on any single transformation in isolation.

### 5.2. Effectiveness of the Proposed Hybrid Model with Transformer Architecture

Experiments II and III further reinforced the effectiveness of the proposed CNN + Transformer model. The Transformer’s self-attention mechanism offers an advantage in modelling temporal dependencies and isolating relevant features within complex signals. Unlike recurrent models (LSTM, GRU), it captures long-range dependencies without recurrence, which is crucial for multi-user activity recognition. In Experiment III, the CNN + Transformer achieved an F1-score of 0.934 in the mixed multi-user scenario, outperforming CNN + GRU (0.928), CNN + BiLSTM (0.930), and CNN + LSTM (0.926). These findings suggest that attention-based modelling enhances temporal resolution and activity discrimination, particularly in noisy multi-user environments. Additionally, its consistent performance across varying augmentation levels and user configurations highlights its generalisability and suitability for deployment in dynamic real-world conditions.

### 5.3. Performance Under HAR Multi-User Configuration

Experiment III focused on assessing the proposed framework’s robustness in multi-user HAR settings, where concurrent activity signals introduce additional complexity due to overlapping motion patterns. Despite these challenges, all hybrid models demonstrated consistently high classification accuracy across two-user and three-user configurations, with only a moderate degradation observed under four-user and mixed-user scenarios. Among the models, the proposed CNN+Transformer performed best. This can be attributed to the global attention mechanism, which proved effective at disentangling simultaneous motion signals by leveraging contextual dependencies across time, enabling the model to distinguish activities even when users performed similar or overlapping actions. In contrast, purely sequential models with LSTM layers exhibited a more notable decline in performance as the number of users increased. This decline can be attributed to their limited ability to capture bidirectional and long-range relationships without explicit architectural modifications.

**Figure 8:**
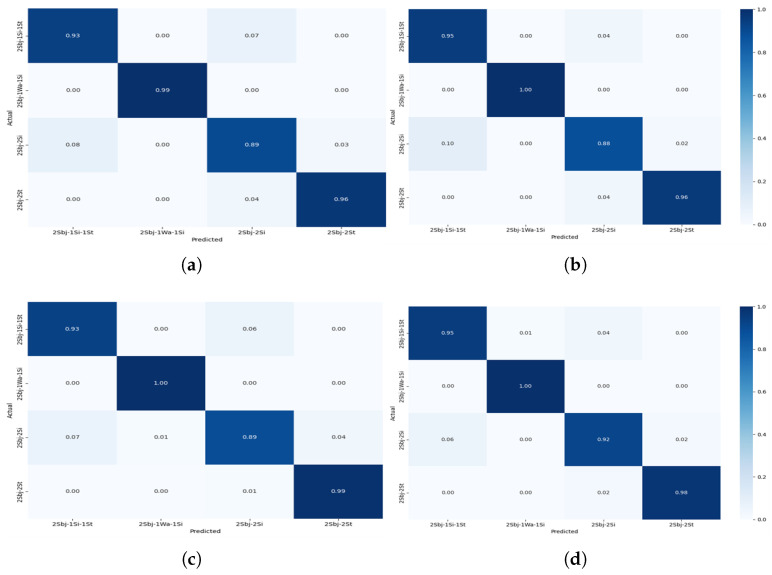
Confusion matrix for Experiment II showing the performance of the models for multi-users—2 subjects for different activities: (**a**) CNN + Transformer (**b**) CNN + GRU (**c**) CNN + BiLSTM (**d**) CNN + LSTM.

**Figure 9:**
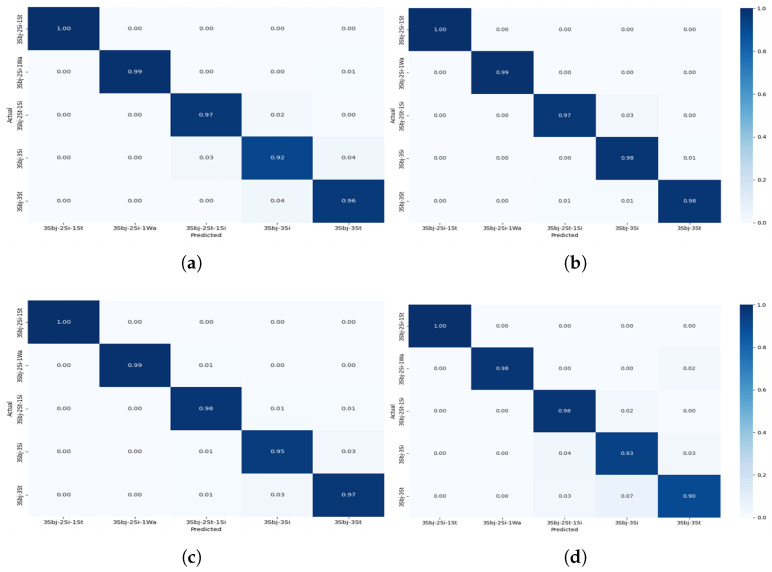
Confusion matrix for Experiment II showing the performance of the models for multi-users—3 subjects for different activities: (**a**) CNN + Transformer (**b**) CNN + GRU (**c**) CNN + BiLSTM (**d**) CNN + LSTM.

**Figure 10:**
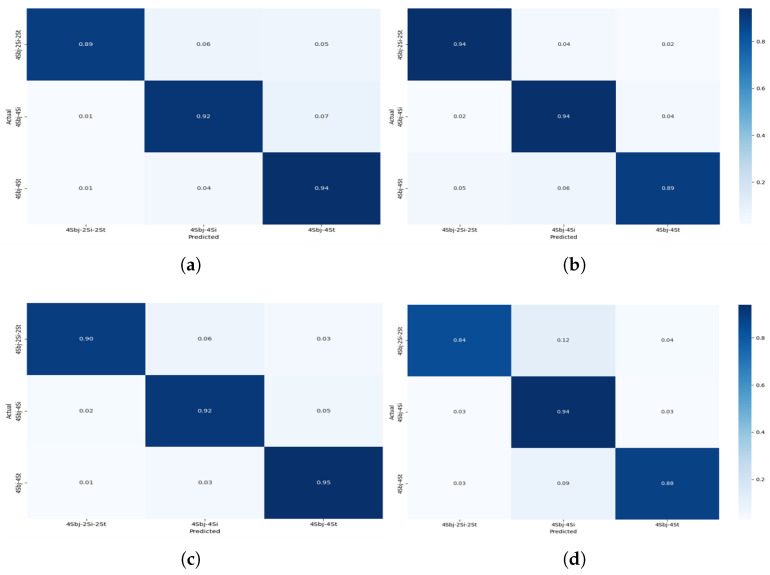
Confusionmatrix for Experiment II showing the performance of the models for multi-users—4 subjects for different activities: (**a**) CNN + Transformer (**b**) CNN + GRU (**c**) CNN + BiLSTM (**d**) CNN + LSTM.

**Figure 11:**
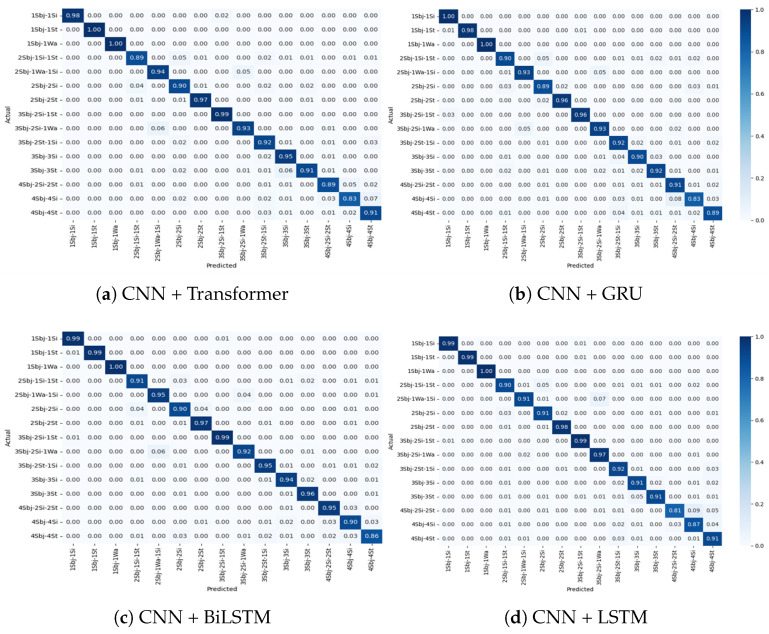
Confusion matrix for Experiment II showing the performance of the models for multiple users—combined 2-3-4 subjects for different activities.

The confusion matrices shown in Figure 8, Figure 9, Figure 10 and Figure 11 provide detailed insights into the per-activity classification performance under varying multi-user settings, revealing important patterns and challenges. In the two-user configuration (Figure 8), the proposed CNN + Transformer model exhibited strong discriminative ability, achieving high precision and recall across most activities. Dynamic activities such as walking were identified with particularly high accuracy, with minimal confusion between them. This is reflective of the distinct temporal patterns which the hybrid model’s CNN and Transformer layers captured effectively.

As the number of concurrent users increased to 3 (Figure 9), classification accuracy remained very good, although slight increases in confusion began to emerge. Specifically, while dynamic activities such as walking continued to be classified correctly for the majority of instances, a marginal rise in misclassification between standing and sitting became apparent. This can be attributed to the overlapping sensor signatures of these low-movement activities, which become increasingly indistinct when recorded from multiple users simultaneously.

The four-user scenario (Figure 10) further amplified these challenges. Static activities, such as standing and sitting, experienced a noticeable drop in classification precision. Standing was frequently misclassified as sitting, and sitting was sometimes confused with especially when two users or one user was involved. This degradation is likely due to the reduced motion variance across users performing sedentary activities at the same time, leading to highly similar sensor patterns that even the attention mechanism found difficult to differentiate. Despite this, the activities continued to be reliably classified, demonstrating the framework’s robustness for dynamic activity detection even in dense user environments.

Under the mixed-user setting, where two, three, and four concurrent users were randomly present (Figure 11), the model maintained good performance, albeit with the most pronounced confusion rates observed across all settings. Again, misclassification was predominantly limited to low-motion activities. For example, confusion between standing and sitting reached its highest rate here, reflecting the real-world complexity when diverse user activities are interleaved. Notably, despite these challenges, the Transformer-based model was able to maintain separation between more kinetically distinct activities, such as walking, demonstrating the capability of capturing global temporal dependencies. These observations suggest that while the Transformer module effectively handles dynamic activity signals under user concurrency, there is room for enhancement when addressing static activities in overlapping scenarios.

**Figure 12:**
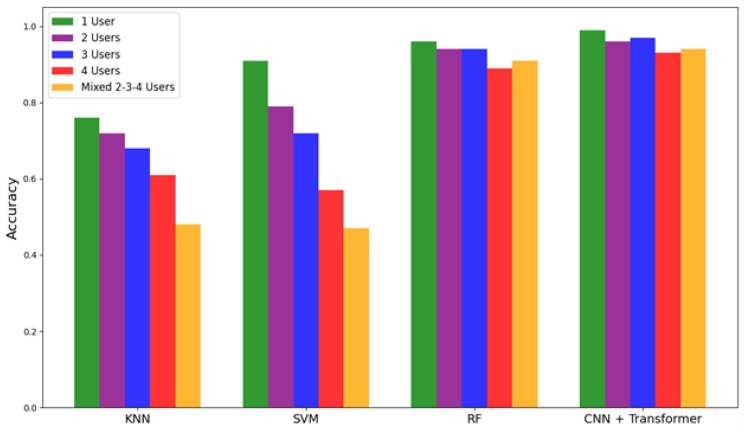
Comparisonof classification accuracy across different classifiers and proposed CNN+Transformer architecture under varying user configurations.

To further assess model efficacy, an additional comparative experiment was conducted using classical machine learning classifiers KNN, SVM, and RF across the same multi-user configurations. The accuracy scores are visualised in Figure 12, with individual bars representing performance across 1-user, 2-user, 3-user, 4-user, and mixed 2-3-4 user setups. It is observed that, in contrast to classical models, CNN + Transformer performed best across all configurations, with performance only marginally affected as user concurrency increased. KNN and SVM, in particular, showed notable declines in accuracy as the number of users increased. This is primarily due to their limited ability to disambiguate overlapping signal sequences in the temporal domain. These results further demonstrate the proposed model’s effectiveness and its suitability for multi-occupant applications where distinguishing between concurrent activity streams is critical.

Further comparative analysis based on Table 8 reinforces these observations. A standalone CNN model was tested, which achieved an accuracy of 89.3%, improving upon prior CNN-only approaches reported in the literature (85.7% [36]). The LSTM model provided a slight improvement to 90.0%, benefitting from its temporal sequence modelling capabilities. However, the proposed CNN+Transformer achieved the highest performance, with an accuracy of 93.9%, an F1-score of 93.3%, precision of 94.0%, and recall of 93.4%.

This significant gain can be attributed to the hybrid model’s ability to integrate spatial feature extraction (via CNN) with long-range temporal dependency learning (via Transformer), offering a balanced and comprehensive representation of user activities. In particular, the high F1-score suggests that the model maintained a strong balance between precision and recall, essential in avoiding both false positives and false negatives in critical HAR applications.

## 6. Conclusions

This paper presents a robust and scalable CSI-based HAR framework tailored for complex multi-user indoor environments. The method integrates multi-stage preprocessing, data augmentation, and a CNN + Transformer hybrid model, alongside other deep learning architectures, to effectively capture spatiotemporal CSI dependencies. Extensive experiments validate the effectiveness of the approach. Sensitivity analysis confirmed that augmentation preserves the underlying data distribution. Moderate augmentation, specifically at factor 5, improved model performance in all variants, with CNN + Transformer consistently outperforming others. The framework demonstrated high adaptability in multi-user experiments, maintaining strong classification accuracy even in dense user configurations. Overall, the proposed CSI-based HAR system exhibits notable improvements in robustness, accuracy, and scalability, forming a strong foundation for future intelligent activity recognition systems in ambient settings. Key limitations include non-adaptive augmentation, lack of explicit user-level separation, and the Transformer’s computational demands. While user density and activity diversity are incorporated into the experimental protocol, the absence of external validation on an independent dataset limits the broader generalisability of the findings. Future work will focus on adaptive augmentation, finer-grained user identification, lightweight Transformer variants, and deployment across diverse real-world environments and populations. We also plan to include deeper multivariate analyses, such as principal component analysis and autocorrelation, to examine the latent structure and the validation of the current model on independently simulated CSI environments to better evaluate cross-environment robustness and strengthen the broader applicability of the proposed system.

## Figures and Tables

**Table 1:** Literature summary table.

Authors (Year)	Dataset/Source	Model(s) Used	Signal Processing/Augmentation	Evaluation Method	Key Findings
Muaaz et al. (2021) [26]	WiFi NICs CSI	CNN	CSI ratio, PCA, spectrogram	Experimental evaluation	97.78% accuracy; robust to environmental variations
Shi et al. (2022) [28]	WiFi CSI	CNN + Domain Adaptation	CSI enhancement	Cross-domain evaluation	One-fits-all model with improved generalisation to new environments
Wang et al. (2022) [29]	WiFi CSI data	Domain Generalisation (AFFAR)	Adaptive feature fusion	Cross-domain testing	Combined domain-specific and domain-invariant features for robustness
Abuhoureyah et al. (2024) [30]	Custom multi-user CSI data	Deep learning + ICA + CWT	ICA, CWT	Experimental evaluation	Separated overlapping signals; enabled robust multi-user location-independent HAR
Wang et al. (2021) [31]	CSI-based HAR dataset	Few-shot Learning	Data augmentation	Experimental evaluation	Few-shot learning enabled improved accuracy in limited-data settings
Zhang et al. (2022) [32]	Custom WiFi CSI	Graph Few-shot Learning	Augmented graph features	Few-shot evaluation	Generalised well across tasks using limited labelled samples effectively
Xiao et al. (2024) [33]	Synthetic WiFi CSI	Diffusion + Contrastive Learning	Diffusion-based augmentation	Contrastive accuracy eval	Outperformed baseline models in generalisation under limited data
Zhang et al. (2022) [34]	CSI-based HAR dataset	Zero-effort cross-domain (Widar3.0)	None specified	Cross-domain evaluation	Achieved high accuracy without requiring user calibration
Shi et al. (2020) [35]	WiFi CSI	One-shot Learning + CSI enhancement	CSI signal denoising	Experimental testing	Enabled recognition with few samples and improved signal quality
Xiao et al. (2023) [33]	WiFi CSI synthetic data	Diffusion Model + MLP	GAN/Diffusion-based augmentation	Comparative experiments	Improved training effectiveness using synthetic CSI data
Elkelany et al. (2023) [27]	CSI dataset (12 activities, 3 environments)	ABiLSTM	Spectrogram conversion	10-fold CV	Achieved up to 94.03% accuracy across environments

**Table 2:** CSI feature categories and descriptions.

Feature Category	Description
Time-Domain Statistical	Mean, Median, Std Dev, Min, Max, Kurtosis, Skewness, IQR, Variance, Root Mean Square
Temporal Dynamics	Mean Absolute Difference, Mean Difference, Median Absolute Difference, Sum of Absolute Differences
Signal Shape Characteristics	Peak-to-Peak Distance, Area Under Curve, Spectral Slope
Frequency Domain	FFT Mean Coefficient, Spectral Centroid (Weighted frequency mean), Spectral Entropy, Spectral Kurtosis
Signal Complexity	Permutation Entropy, Weighted Permutation Entropy, Spectral Variation (Normalised spectral std dev)
Energy/Power Features	Absolute Energy, Average Power, Wavelet Energy (Sum of squared DWT coefficients)
Statistical Distribution	Mean Absolute Deviation, Median Absolute Deviation, ECDF Percentile (25th percentile)
Spectral Relationships	Power Bandwidth (Integrated spectrum), Spectral Distance (Cumulative spectral differences)

**Table 3:** Hyperparameter settings for the CNN + Transformer model.

Component	Hyperparameter	Value
Architecture	Number of Conv1D Layers	3
	Conv1D Filter Sizes	32, 64, 128
	Kernel Size	3
	Pooling Type	MaxPooling1D, GlobalMaxPooling1D
	Batch Normalisation	Yes
Transformer	Encoder Layers	3
	Attention Heads	4
	Head Size	64
	Feedforward Dimension	256
	Dropout Rates	0.1 (Transformer), 0.5, 0.3 (Dense)
	Positional Encoding	Relative (vector-based)
Training	Optimiser	Adam
	Learning Rate	$1\times 10^{-3}$
	Batch Size	32
	Epochs	500
	Early Stopping Patience	10
	LR Schedule	Step decay at epochs 20 and 30
	Loss Function	Sparse Categorical Cross-entropy

**Table 4:** Mann–Whitney U and Levene’s test results for original vs. augmented data.

Feature	Label	Mann–Whitney U	*p*-Value	Levene’s W	*p*-Value	Interpretation
Mean	Empty	144,584.0	0.9139	0.1772	0.6739	Fail to reject $H_{0}$
Mean	1Subject-1Sit	206,671.5	0.9325	0.0449	0.8322	Fail to reject $H_{0}$
Mean	2Subjects-1Sit-1Stand	105,480.5	0.9384	0.0819	0.7747	Fail to reject $H_{0}$
Mean	3Subjects-2Sit-1Stand	105,100.5	0.9871	0.0858	0.7696	Fail to reject $H_{0}$
Mean	4Subjects-2Sit-2Stand	106,725.5	0.7811	1.0095	0.3151	Fail to reject $H_{0}$

**Table 5:** Experiment II results: accuracy across different augmentation factors for each model and phase.

Model	Aug. Factor	Phase 1	Phase 2	Phase 3	Phase 4	All Activities
CNN + Transformer	0	0.869	0.840	0.899	0.869	0.757
	1	0.963	0.927	0.957	0.885	0.817
	3	0.988	0.970	0.976	0.938	0.910
	5	0.994	0.979	0.987	0.959	0.939
	7	0.994	0.986	0.990	0.965	0.954
	10	0.994	0.989	0.991	0.973	0.963
CNN + BiLSTM	0	0.807	0.852	0.885	0.871	0.766
	1	0.968	0.925	0.957	0.874	0.805
	3	0.987	0.968	0.974	0.933	0.908
	5	0.992	0.977	0.986	0.955	0.937
	7	0.992	0.984	0.990	0.964	0.948
	10	0.995	0.985	0.992	0.972	0.960
CNN + GRU	0	0.762	0.729	0.832	0.881	0.772
	1	0.965	0.932	0.960	0.875	0.799
	3	0.985	0.969	0.979	0.935	0.906
	5	0.993	0.978	0.985	0.954	0.930
	7	0.993	0.985	0.989	0.963	0.952
	10	0.994	0.987	0.991	0.973	0.958
CNN + LSTM	0	0.746	0.729	0.841	0.869	0.761
	1	0.962	0.927	0.952	0.872	0.794
	3	0.984	0.966	0.974	0.933	0.901
	5	0.990	0.973	0.983	0.954	0.927
	7	0.993	0.982	0.988	0.965	0.949
	10	0.995	0.985	0.989	0.973	0.959

**Table 6:** Experiment III results: multi-user presence and activity detection.

Model	Metric	One User	Two Users	Three Users	Four Users	Mixed 2/3/4 Users
CNN + Transformer	Accuracy	0.997	0.956	0.971	0.923	0.938
	Precision	0.997	0.956	0.970	0.923	0.935
	Recall	0.997	0.955	0.971	0.923	0.934
	F1-score	0.997	0.955	0.971	0.923	0.934
CNN + BiLSTM	Accuracy	0.995	0.953	0.971	0.916	0.935
	Precision	0.995	0.953	0.971	0.917	0.930
	Recall	0.995	0.953	0.970	0.917	0.930
	F1-score	0.995	0.953	0.971	0.917	0.930
CNN + GRU	Accuracy	0.996	0.952	0.971	0.921	0.933
	Precision	0.996	0.952	0.970	0.921	0.930
	Recall	0.996	0.952	0.970	0.920	0.929
	F1-score	0.996	0.952	0.970	0.920	0.928
CNN + LSTM	Accuracy	0.995	0.953	0.961	0.896	0.930
	Precision	0.994	0.953	0.960	0.896	0.926
	Recall	0.995	0.952	0.960	0.895	0.926
	F1-score	0.995	0.942	0.960	0.895	0.926

**Table 7:** Performance of CNN+Transformer with individual and cumulative augmentations (augmentation factor = 5) across all activity classes.

Model Variant	Accuracy (%)	F1-Score	Precision	Recall
CNN + Transformer + No Aug.	0.872	0.890	0.891	0.893
+ Magnitude_Warp	0.837	0.819	0.823	0.819
+ Magnitude_Warp + Slice_Shuffle	0.907	0.900	0.901	0.900
+ Magnitude_Warp + Slice_Shuffle + Scale	0.928	0.922	0.923	0.922
+ Magnitude_Warp + Slice_Shuffle + Scale +Jitters	0.939	0.933	0.940	0.934

**Table 8:** Comparison of classification performance across different model architectures, including standalone CNN, LSTM, and the proposed CNN + Transformer.

Model Variant	Accuracy	F1-Score	Precision	Recall
CNN [36]	0.857	–	–	–
CNN	0.893	0.891	0.893	0.892
LSTM	0.900	0.894	0.894	0.894
Proposed CNN + Transformer	0.939	0.933	0.940	0.934

## Data Availability

The dataset for this study is publicly available.
